# Rib hump deformity assessment using the rib index in adolescent scoliotics treated with full screw or hybrid constructs: aetiological implications

**DOI:** 10.1186/1748-7161-10-S1-O48

**Published:** 2015-01-19

**Authors:** Konstantinos C Soultanis, Nikolaos A Stavropoulos, Theodoros B Grivas, Konstantinos Tsiavos, Konstantinos Starantzis, Panayiotis J Papagelopoulos

**Affiliations:** 1 1st Orhopaecic Department, Athens University, Medical School, Greece; 2 Department of Orthopaedics and Traumatology, TZANEIO General Hospital of Piraeus, Piraeus, Greece

## Objectives

Idiopathic scoliosis (IS) is a 3D deformity of the trunk with detectable bony asymmetries and deformities not only of the spine - the central axis - but also of the rib cage and other parts of the body. The thorax, like any other part of the skeleton displays variations in dimensions and proportions. The bony framework of the thorax changes so that the side to side width of the chest cavity exceeds the anteroposterior length while the ribs become structurally stronger as the child grows up. Review of literature reveals that in Idiopathic Scoliosis (IS) surgery, the post-op correction of the thoracic deformity using full transpedicular screw and hybrid constructs, applying the Rib-Index (RI) method, was never compared. Therefore the aim of this report is to study which of the above two constructs offers better postoperative RH deformity correction.

## Material and methods

Sixteen children with Adolescent Idiopathic Scoliosis (AIS) were operated upon using full pedicle screw (group A) while nine using hybrid construct (group B). The median age for group A was 15 years (range, 10 to 35 years) and for group B 17.2 years. The RH deformity was assessed on the lateral spinal radiographs using the RI, as it was introduced by Grivas et al 2002. Moreover the amount of RI Correction was calculated by subtracting the postoperative RI from the preoperative RI. The correction percentage was calculated using the equation: RI Correction (%) = [(Preoperative RI-Postoperative RI) / Preoperative RI] x 100. Statistical analysis was done using the Wilcoxon test - SPSSv.20 package.

## Results

In group A the mean pre-op RI was 1.93 and the post-op 1.37 (p<0.001). Similarly in group B the mean pre-op RI was 2.06 while the mean post-op 1.51, (p=0.008). However, between group A and B the RI correction means was found to be no statistically significant, p=0.803.

## Conclusions

Although the pre and postoperative RI correction was significant within the two groups, this did not happen among the two groups. It appears that the RH deformity correction is not different no matter the spinal construct type used. Provided that the full screw construct is powerful, the post-op derotation and RH deformity correction would be better than when a hybrid construct is applied, which is not the case in this study. This ribcage discordance behavior from spine after surgery could be attributed to a different scoliogenic course and this could have potential aetiological implications.

